# When Bullous Pemphigoid Is Not Bullous Pemphigoid: The Importance of Going Beyond Direct Immunofluorescence

**DOI:** 10.7759/cureus.22201

**Published:** 2022-02-14

**Authors:** Christina R Hopkins, Vicky Ren, Raminder Grover, Clay Cockerell, Sylvia Hsu

**Affiliations:** 1 Dermatology, Baylor College of Medicine, Houston, USA; 2 Laboratory Medicine, Beutner Laboratories, Buffalo, USA; 3 Dermatology, University of Texas Southwestern Medical Center, Dallas, USA; 4 Dermatology, Temple University Hospital, Philadelphia, USA

**Keywords:** salt-split skin, indirect immunofluorescence, direct immunofluorescence, autoimmune bullous disease, bullous pemphigoid, laminin gamma-1, anti-p105 pemphigoid, anti-p200 pemphigoid

## Abstract

Bullous pemphigoid (BP) is the most common autoimmune bullous disease, but rarer forms of pemphigoid may appear identical to BP on routine histopathology and direct immunofluorescence (DIF). Here, we present the case of a 60-year-old man, who was initially thought to have BP, with supportive findings on routine histopathology and DIF. However, prominent oral involvement and cutaneous lesions refractory to conventional treatment suggested an alternate diagnosis. Further workup was performed, including indirect immunofluorescence (IIF) on salt-split skin, which showed binding of antibodies to the dermal floor rather than to the blister roof, and enzyme-linked immunosorbent assay for pemphigus and pemphigoid antibodies. With these additional tests, we concluded that the patient does not have BP but rather anti-p200 pemphigoid, anti-p105 pemphigoid, or a yet undiscovered form of pemphigoid. We reached a presumptive diagnosis of anti-p200 pemphigoid, as it is the most common pemphigoid with serum antibodies to the dermal floor of human salt-split skin by IIF. This case demonstrates that suspicion for other autoimmune bullous diseases in cases of treatment-refractory and clinically aberrant BP is essential. A limited workup may lead to a missed diagnosis and ultimately less efficient disease management.

## Introduction

Bullous pemphigoid (BP), the most common autoimmune bullous disease (AIBD), features eosinophilic spongiosis or a subepidermal blister with eosinophils on routine histopathology and immunoglobulin G (IgG) and/or C3 basement membrane zone (BMZ) deposition on direct immunofluorescence (DIF) [1]. In cases where BP is refractory to treatment, the clinician should suspect rarer forms of pemphigoid, which can appear identical to BP on routine histopathology and DIF. Further workup is necessary in these instances [2-3]. Here, we present a case of presumptive anti-p200 pemphigoid and the diagnostic approach utilized.

## Case presentation

**Figure 1:**
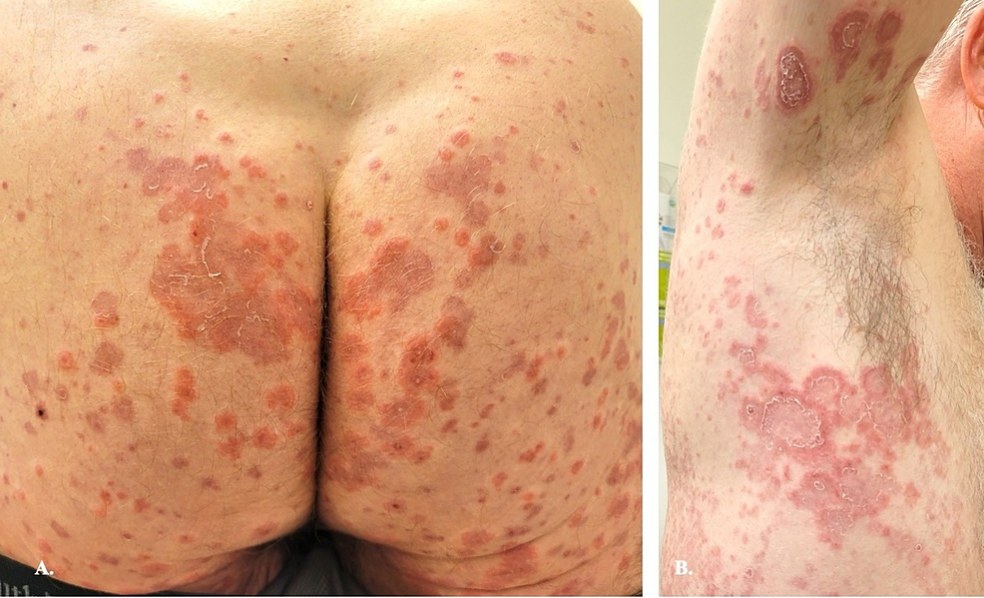
Anti-p200 pemphigoid; clinical photos A&B: Erythematous papules and vesicles coalescing into scaly, annular, polycyclic plaques on the buttocks and posterior thighs, axillae, and flanks

**Figure 2:**
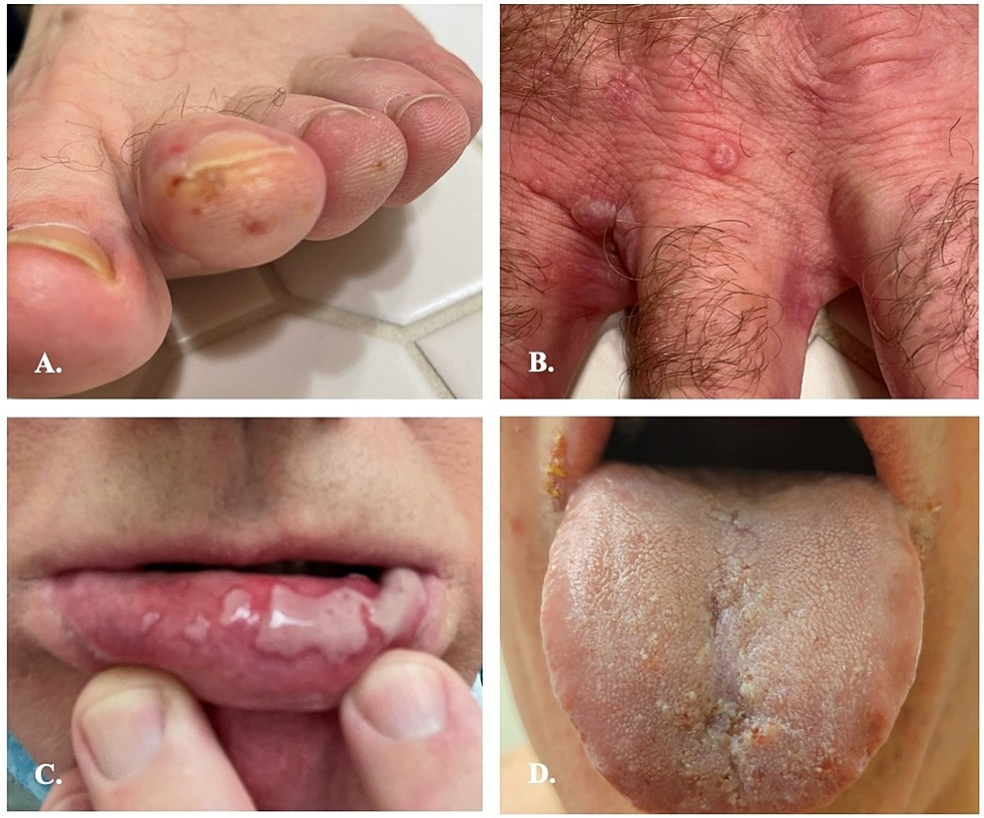
Anti-p200 pemphigoid; clinical photos A. Vesicles on the distal toes; B. Papulovesicles on the dorsal hand and interdigital spaces; C. Vesiculobullous lesions on the inferior vermillion lip; D: Erosions and vesicles on the distal dorsal tongue

A 60-year-old man presented for evaluation of a generalized, blistering rash. Physical examination showed erythematous papules and vesicles - some in an annular, herpetiform arrangement - on the trunk, axillae, upper extremities (including palms and fingers), buttocks, inner thighs, and toes, as well as ulcers on the vermilion lips, tongue, and palate (Figures 1A-1B, Figures 2A-2D).

An outside dermatologist had performed punch biopsies for routine histopathology and DIF on two separate occasions. Initial histopathology showed a subepidermal blister with neutrophils concerning for dermatitis herpetiformis (DH) or bullous systemic lupus erythematosus (SLE) while DIF showed a granular, linear band of IgG and C3 at the dermal-epidermal junction (DEJ) concerning for an unusual BP. However, subsequent histopathology and DIF were both consistent with BP. The patient trialed oral dapsone without improvement and had been on prednisone 50 mg daily for over a month with persistent flaring.

**Figure 3:**
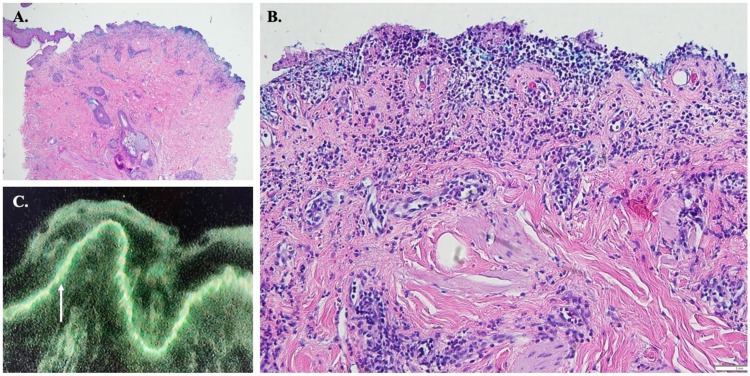
Anti p-200 pemphigoid; routine histopathology and direct immunofluorescence A&B: Subepidermal blister with mixed dermal infiltrate of lymphocytes, histiocytes, and eosinophils (hematoxylin-eosin stain; x4 and x20 magnification, respectively); C: Linear band of IgG and C3 at the dermal-epidermal junction (white arrow) with n-serration (direct immunofluorescence; x20 magnification)

We started the patient on dupilumab while continuing prednisone; however, the significant oral involvement and recalcitrant cutaneous lesions suggested an AIBD other than BP, warranting further workup. Repeat biopsies showed a subepidermal vesicle with a mixed dermal infiltrate of lymphocytes, histiocytes, and eosinophils and a smooth band of IgG and C3 with an n-serration pattern along the DEJ, consistent with BP (Figures 3A-3C).

**Figure 4:**
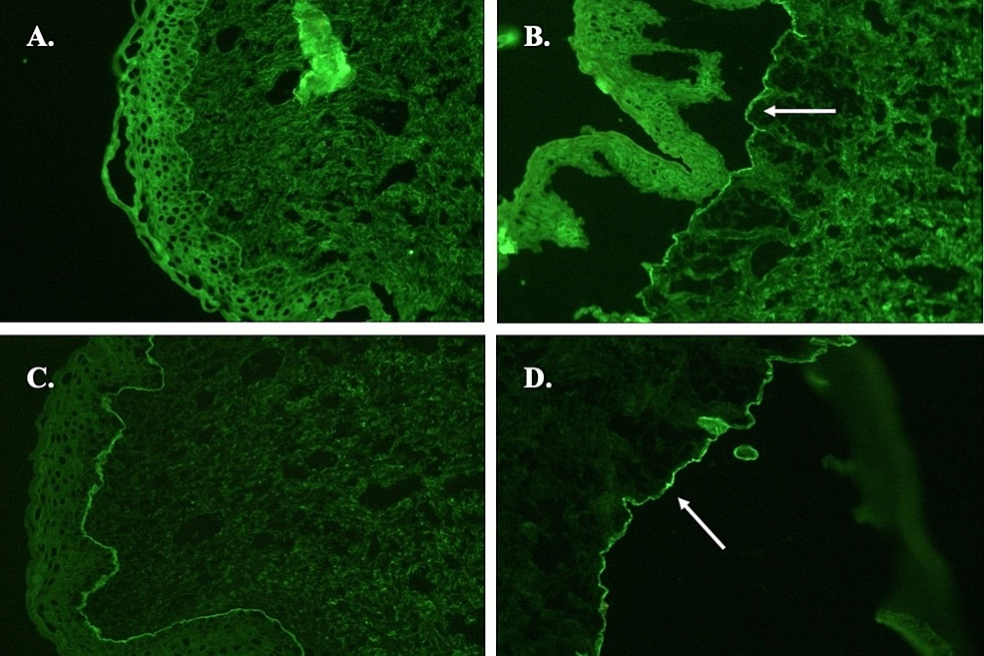
Anti-p200 pemphigoid; indirect immunofluorescence on salt-split skin A: IgG unsplit skin; B: IgG on 1.0 M NaCl split skin; C: IgG4 unsplit skin; D: IgG4 on 1.0 M NaCl split skin. IgG and IgG4 basement membrane zone antibodies binding to the dermal floor on salt-split skin (white arrows) IgG: immunoglobulin G

However, enzyme-linked immunosorbent assay (ELISA) for BP antibodies did not correlate with the extent of clinical disease: BP180 was low-positive (17.1U/ml; reference: negative <9U/ml) and BP230 was normal. Antinuclear antibody was negative, as was ELISA for antibodies to desmogleins 1 and 3, envoplakin 3, and type VII collagen, thus ruling out paraneoplastic pemphigus (PNP), pemphigus vulgaris (PV), pemphigus foliaceus (PF), epidermolysis bullosa acquisita (EBA), and bullous SLE. Indirect immunofluorescence (IIF) on the monkey esophagus and rat bladder epithelium was negative, thus eliminating PV and PNP, respectively. IIF on salt-split skin showed strong binding of IgG and IgG4 BMZ antibodies to the dermal floor (Figures 4A-4D), as is seen with EBA, anti-laminin 332 mucous membrane pemphigoid (MMP), anti-p105 pemphigoid, and anti-p200 pemphigoid.

In the United States, there are currently no commercially available tests for anti-laminin 332, anti-p105, or anti-p200 (laminin gamma-1). However, research testing for laminin 332 antibodies by IIF was negative for IgG and IgG4.

By this process of exclusion, we concluded that the patient does not have BP but rather anti-p200 pemphigoid, anti-p105 pemphigoid, or a yet undiscovered form of pemphigoid. We favor a diagnosis of anti-p200 pemphigoid, as it is the most common pemphigoid with serum antibodies to the dermal floor of human salt-split skin by IIF [4]. Furthermore, compared to BP, anti-p200 pemphigoid exhibits prominent mucosal, palmoplantar, and cephalic involvement and an earlier age of onset (mean age ~65 in anti-p200 pemphigoid versus 70+ in BP), as seen in our patient [3]. Anti-p105 pemphigoid is less likely, as it clinically resembles toxic epidermal necrolysis or PV; routine histopathology shows a predominately neutrophilic infiltrate, and DIF resembles that of BP [5].

The patient continued to flare uncontrollably with dupilumab, which was discontinued in favor of an increase to prednisone 100 mg daily, two infusions of rituximab 1000 mg spaced two weeks apart, and initiation of dapsone 100 mg daily. He has experienced promising improvement and is now on prednisone 60 mg daily one month after the second dose of rituximab.

## Discussion

Rare forms of pemphigoid, including anti-p200 pemphigoid, may present a diagnostic challenge to clinicians, mimicking a variety of more common AIBDs both clinically and histopathologically [3]. A 2019 systematic review of 113 anti-p200 pemphigoid patients found that the disease most often clinically resembles BP (66% cases) and rarely mimics other AIBDs such as linear IgA bullous dermatosis, EBA, DH, and MMP [3]. Prominent mucosal, palmoplantar, and cephalic involvement should heighten suspicion for anti-p200 pemphigoid, and scarring and milia are observed in a minority of patients (~15% cases) [3]. An association with psoriasis has also been reported [3].

Biopsy of anti-p200 pemphigoid typically reveals a subepidermal blister with a neutrophilic or mixed inflammatory infiltrate, although papillary microabscesses and a neutrophilic or eosinophilic spongiotic component may be present [6-7]. A linear band of IgG and/or C3 with an n-serrated pattern on DIF is characteristic [2-3,7]. The u-serrated pattern is unique to anti-type VII collagen diseases (i.e., EBA and bullous SLE), highlighting the localization of type VII collagen to the sublamina densa [8]. The n-serration pattern seen in other forms of pemphigoid, including anti-p200 pemphigoid, corresponds to targets within or superior to the lamina densa [8-9].

IIF on salt-split skin helps distinguish anti-p200 pemphigoid from BP and most forms of MMP. Dermal (floor) localization is observed in the former and epidermal (roof) localization in the latter two entities [3,10]. As anti-laminin 332 MMP, anti-p200 pemphigoid, and presumably anti-p105 pemphigoid (given its antigen location in the lower lamina lucida) all demonstrate an n-serration pattern on DIF and dermal binding on IIF, further testing is required to distinguish these entities [8]. However, due to the complexity of workup and lack of access to confirmatory tests, it is likely that they have been widely underdiagnosed [3,7].

## Conclusions

This case demonstrates that despite supportive histopathology and DIF, suspicion for another AIBD is necessary in cases of treatment-refractory and clinically aberrant BP. IIF on salt-split skin can assist in differentiating BP from rarer forms of pemphigoid. Furthermore, examination of the serration pattern on DIF can aid in distinguishing anti-type VII collagen disorders from other subepidermal bullous diseases. Clinicians should be aware that limited workup may lead to a missed diagnosis and less efficient management of the actual disease.
